# An Investigation of Compensation and Adaptation to Auditory Perturbations in Individuals With Acquired Apraxia of Speech

**DOI:** 10.3389/fnhum.2018.00510

**Published:** 2018-12-19

**Authors:** Kirrie J. Ballard, Mark Halaki, Paul Sowman, Alise Kha, Ayoub Daliri, Donald A. Robin, Jason A. Tourville, Frank H. Guenther

**Affiliations:** ^1^Faculty of Health Sciences, University of Sydney, Lidcombe, NSW, Australia; ^2^Department of Cognitive Sciences, Macquarie University, Sydney, NSW, Australia; ^3^Department of Speech and Hearing Science, Arizona State University, Tempe, AZ, United States; ^4^Department of Communication Sciences and Disorders, Interdisciplinary Program in Neuroscience and Behavior, University of New Hampshire, Durham, NH, United States; ^5^Department of Speech, Language and Hearing Sciences, Boston University, Boston, MA, United States

**Keywords:** feedback, auditory perturbation, pitch, F0, formant frequency, speech, motor control, apraxia of speech

## Abstract

Two auditory perturbation experiments were used to investigate the integrity of neural circuits responsible for speech sensorimotor adaptation in acquired apraxia of speech (AOS). This has implications for understanding the nature of AOS as well as normal speech motor control. Two experiments were conducted. In Experiment 1, compensatory responses to unpredictable fundamental frequency (F0) perturbations during vocalization were investigated in healthy older adults and adults with acquired AOS plus aphasia. F0 perturbation involved upward and downward 100-cent shifts versus no shift, in equal proportion, during 2 s vocalizations of the vowel /a/. In Experiment 2, adaptive responses to sustained first formant (F1) perturbations during speech were investigated in healthy older adults, adults with AOS and adults with aphasia only (APH). The F1 protocol involved production of the vowel /ε/ in four consonant-vowel words of Australian English (*pear, bear, care, dare*), and one control word with a different vowel (*paw*). An unperturbed Baseline phase was followed by a gradual Ramp to a 30% upward F1 shift stimulating a compensatory response, a Hold phase where the perturbation was repeatedly presented with alternating blocks of masking trials to probe adaptation, and an End phase with masking trials only to measure persistence of any adaptation. AOS participants showed normal compensation to unexpected F0 perturbations, indicating that auditory feedback control of low-level, non-segmental parameters is intact. Furthermore, individuals with AOS displayed an adaptive response to sustained F1 perturbations, but age-matched controls and APH participants did not. These findings suggest that older healthy adults may have less plastic motor programs that resist modification based on sensory feedback, whereas individuals with AOS have less well-established and more malleable motor programs due to damage from stroke.

## Introduction

Acquired apraxia of speech (AOS) is a disorder of speech motor control subsequent to damage in the left inferior frontal cortex, particularly the ventral premotor cortex (vPMC), in adults who previously had normal speech production (Robin et al., 2008; Ziegler, 2008; Duffy, 2013; Ballard et al., 2014; New et al., 2015). According to the Directions into Velocities of Articulators (DIVA) model of speech motor control (Guenther et al., 1998; Guenther, 2006, 2016; Guenther and Vladusich, 2012), generation of articulatory movements involves integration of two control systems: *feedforward* and *feedback control*, with the latter involving auditory and somatosensory subsystems. The model proposes that left vPMC is crucial for the readout of finely tuned speech motor programs for frequently produced speech sequences such as syllables from the native language; these motor programs constitute the feedforward component of speech motor commands. Damage to left vPMC impairs the readout of these motor programs, resulting in the primary symptoms that characterize AOS (see also Ballard and Robin, 2007; Maas et al., 2015).

Although some past studies suggest that auditory feedback control mechanisms may be intact in AOS, the situation is less clear than for feedforward control mechanisms that are widely believed to be impaired in AOS. Past studies have relied primarily on masking noise to investigate auditory feedback control mechanisms in AOS, and these studies have produced mixed results. Jacks and Haley (2015) found that masking noise increased fluency in some AOS patients, suggesting that auditory feedback control mechanisms may actually impair the speech of these AOS patients when normal auditory feedback is present. Maas et al. (2015) found that masking noise led to a reduction in vowel contrasts that was greater for AOS participants than for healthy controls, suggesting that auditory feedback control may help AOS patients produce more distinct vowels when auditory feedback is present (see also Iuzzini-Seigel et al., 2015). When viewed within the DIVA model framework, although basic auditory feedback control mechanisms (such as those used to maintain a constant pitch or F0) might survive left vPMC damage, such damage could impair the readout of auditory expectations, or “targets,” for ongoing speech sequences to the auditory and somatosensory cortical areas. These targets are crucial for feedback control of segmental parameters such as formant frequencies since they allow for detection of mismatches between expected and actual sensory consequences that drive corrective movements.

In this study, we aim to investigate the degree to which feedforward and auditory feedback control mechanisms are impaired in AOS. Feedforward and feedback control mechanisms for speech are often investigated using perturbations to the speech articulators or acoustic signal during speech. Unexpected perturbations are used to highlight feedback control mechanisms since such perturbations induce sensory errors that are transformed into corrective motor commands for the ongoing production. For example, applying an unexpected load to the lip or jaw during speech results in somatosensory errors that are corrected by the somatosensory feedback control subsystem, which detects these errors and translates them into corrective movements of the lip/jaw system (e.g., Gracco and Abbs, 1985; de Miranda Marzullo et al., 2010). We will refer to these corrective movements to unexpected perturbations, which occur within the same trial as the perturbation, as *compensations*. Similarly, perturbing auditory parameters results in compensatory movements generated by the auditory feedback control system (e.g., Elman, 1981; Burnett et al., 1997; Larson et al., 2001; Purcell and Munhall, 2006a,b; Tourville et al., 2008; Cai et al., 2010, 2011; Flagmeier et al., 2014). In healthy young adults, a 100 cent (i.e., one semitone) shift in the fundamental frequency (F0) of the voice upward or downward typically causes the speaker to change their F0 about 18–20 cents in the opposite direction (e.g., Burnett et al., 1998), with a response latency between 100 and 200 ms (Larson et al., 2000; Hain et al., 2001; Parkinson et al., 2013). Age significantly affects the magnitude of the response, with healthy older adults (60–73 years) producing a larger compensatory response of ∼35 cents away from baseline for a 100 cent shift (Liu et al., 2010); however, older and younger adults have similar response latency (Liu et al., 2010). To date, the integrity of the compensatory response to F0 perturbation in AOS has not been tested (but see Behroozmand et al., 2018). If AOS primarily affects feedforward control, then these patients should demonstrate a compensatory response to unexpected auditory perturbations (which highlight feedback control mechanisms rather than feedforward mechanisms) that is similar to older healthy controls.

If an auditory or somatosensory perturbation is sustained over many trials, more long-lasting *adaptive* (i.e., learned) responses are seen (Houde and Jordan, 1998, 2002; Jones and Munhall, 2000, 2002; Purcell and Munhall, 2006a; Villacorta et al., 2007; MacDonald et al., 2010; Katseff et al., 2012; Rochet-Capellan et al., 2012; Max and Maffett, 2015; Vaughn and Nasir, 2015). These learned responses are identified either by interspersing trials with masking noise within the perturbed feedback trials (e.g., Houde and Jordan, 1998; Villacorta et al., 2007), or by abruptly removing the perturbation after a series of perturbed trials (e.g., Abur et al., 2018). Both methods basically eliminate online compensatory responses since the speakers do not hear a perturbation because auditory feedback is either masked or no perturbation is applied. Thus, any residual “compensatory” response (when there is no perturbation to compensate for on the current trial) can be attributed to adaptive processes that have modified the feedforward commands (or motor programs) for producing the test stimuli. We will refer to these learned responses, which carry over into future productions even if those productions involve masking of feedback or contain no perturbation, as *adaptations* to differentiate them from online compensatory responses.

Sustained auditory perturbation typically involves shifting the first formant (F1) of specific vowels in the acoustic signal. F1 maps tongue height in the oral cavity such that an upward perturbation (i.e., increase in F1 frequency) during production of the vowel /ε/, for example, is interpreted as a drop in tongue height (i.e., toward /a/). This leads the speaker to oppose the perturbation by elevating the tongue and shifting production toward /i/, with the response typically being 10–50% of the magnitude of the original perturbation (e.g., Houde and Jordan, 2002; Purcell and Munhall, 2006a,b; Berry et al., 2014). Presenting the perturbation repeatedly over many trials invokes both a short-term compensatory response as well as a longer-term adaptive response as the speaker adjusts feedforward motor programs to accommodate the repeated error signals. Adaptation is evident in speakers regardless of whether they are aware of the perturbation (Niziolek and Guenther, 2013).

While studies of compensation and adaptation to perturbations are well established in healthy young speakers, the impact of neurological damage to the cortical speech network on feedback and feedforward control processes is not well understood. Furthermore, very few auditory perturbation studies have been performed on healthy adults over 50 years of age (though see Liu et al., 2012; Mollaei et al., 2013, 2016), whereas the large majority of AOS cases involve adults over 50. Further, AOS is suited for investigation as it has been associated with damage to the left inferior frontal cortex (including left vPMC), an area that is thought to be crucially involved in both feedforward and feedback control mechanisms. We hypothesize that the individuals with AOS will demonstrate impaired feedforward control relative to age-matched control participants, with attenuated or absent adaptation to a repeated F1 perturbation. We also investigate auditory feedback control mechanisms in AOS using a pitch perturbation protocol that is the “industry standard” but has not yet been done with individuals with AOS to our knowledge.

Here, we describe an F0 unexpected perturbation study (Experiment 1) and an F1 sustained perturbation study (Experiment 2) aimed at testing the DIVA model hypotheses regarding feedforward and feedback control mechanisms in AOS laid out above. Healthy older adults and adults with acquired AOS participated in a single testing session that included diagnostic testing followed by Experiments 1 and 2. The order of the two experiments was randomized across participants. Given that adults with AOS typically have some degree of co-existing aphasia, we also recruited a group of patients with aphasia only (APH, mixed types) to the F1 perturbation study (Experiment 2) to test whether any differences might be due to a general effect of neurological damage.

## Experiment 1: F0 Perturbation During Vocalization

### Methods

#### Participants

Participants were 12 individuals diagnosed with AOS plus aphasia (AOS; eight males, four females; *M* = 63.3 years, *SD* = 9.1 years, range 50–80 years) secondary to single left hemisphere middle cerebral artery stroke and 10 age-matched healthy older control adults (CTL; six males, four females; *M* = 64.8 years, *SD* = 10.5 years, range 45–79 years). Diagnosis of AOS was based on consensus between two expert judges, using diagnostic criteria of Duffy (2013) as well as meeting both the criteria of Ballard et al. (2016)—a score >0.17 for the Errors on Words of Increasing Length measure and <112 for the Pairwise Variability Index for weak–strong polysyllabic words (see Ballard et al., 2016 for details). Demographic data and results of diagnostic testing for patients are reported in Table 1. Note that AOS092 scored in the normal range on the revised Western Aphasia Battery (Kertesz, 2006) but demonstrated frank word finding difficulty in multiple tasks and so is categorized here as anomic. MRI data were not available for a sufficient number of participants on the same MRI scanner to permit brain-behavioral correlational analyses.

**Table 1:** Demographic and diagnostic testing for participants with apraxia of speech (AOS^∗^) plus aphasia who completed the F0 perturbation study (*N* = 12) and the F1 perturbation study (*N* = 8, in bold font).

ID	Sex	Age (years)	Years post-stroke	WAB-AQ (/100)	Aphasia type	PALPA (/72)	E_WIL	PVI_WS
AOS21	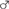	57	16	22.7	Broca	58	1.00	7.0
**AOS22**	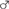	71	12	75.3	Anomic	60	0.25	101.3
AOS30	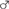	80	14	39.6	Broca	63	1.00	4.8
**AOS49**	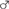	61	4	64.8	Transcortical motor	68	0.47	90.3
**AOS60**	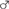	54	7	38.2	Broca	66	0.95	45.1
**AOS77**	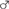	59	4	68.3	Broca	69	0.50	63.9
AOS86	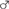	61	4	34.8	Broca	70	0.46	66.0
**AOS92**	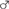	55	5	98.6	Anomic	71	0.19	40.7
**AOS24**	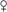	67	10	88.0	Anomic	68	0.42	30.3
**AOS79**	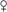	60	3	55.6	Broca	71	0.47	111.4
**AOS88**	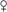	76	3	88.9	Anomic	22	0.25	50.9
AOS89	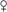	50	7	23.6	Broca	NA	1.00	60.7

	8 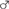	*M* = 62.6	*M* = 7	*M* = 58.2		*M* = 62.4	*M* = 0.58	*M* = 56.0
	4 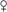	*SD* = 9.1	*SD* = 4.5	*SD* = 26.4		*SD* = 14.1	*SD* = 0.32	*SD* = 33.8


*^∗^AOS diagnosis made by expert judgment and confirmed by both an E_WIL score > 0.17 *and* PVI_WS score < 112 (Ballard et al., 2016); WAB-AQ, Aphasia Quotient from the Western Aphasia Battery Revised (Kertesz, 2006), an index of aphasia severity; PALPA, Psycholinguistic Assessments of Language Processing in Aphasia (Kay et al., 1992); E_WIL, Errors on Words of Increasing Length (Ballard et al., 2016) calculated from the Words of Increasing Length subtest of the Apraxia Battery for Adults – 2 (Dabul, 2000); PVI_WS, pairwise variability index for vowel duration in weak–strong stressed three syllable words (e.g., “banana”; Ballard et al., 2016); NA, not able to do task. Note that participants AOS22, 30, 49 were reported in New et al. (2015) and AOS21 – 77 were reported in Ballard et al. (2016).*

Healthy adults were fluent speakers of Australian English with no self-reported history of speech, language, hearing or neurological disorders, or substance abuse. All scored ≥ 29/30 on the Mini-Mental State Examination (Folstein et al., 1975).

All participants passed a pure-tone screening at 25 dB HL in at least one ear at frequencies of 500, 1,000, and 2,000 Hz (Gates and Hoffman, 2007), demonstrating adequate hearing of the first two formants (i.e., F1 and F2) for the target vowels in the study.

All participants were recruited by on-campus advertisement, from the universities’ registries of healthy controls and communication-impaired stroke cases. All procedures were approved by the Human Research Ethics Committees of Sydney South West Area Health Service, University of Sydney, and Macquarie University, Australia. All procedures conformed to the Declaration of Helsinki (BMJ 1991; 302: 1194). All participants provided written informed consent.

#### Procedure

Participants attended a single testing session that included diagnostic testing followed by Experiments 1 and 2, in random order. Participants were seated in a sound-attenuated booth in front of a computer monitor. They were told that “ah” would appear on the screen and they were to produce that vowel sound until the word disappeared (2 s), take a breath and be ready for the next stimulus (∼2 s interval). They were asked to maintain an even habitual pitch, clear vocal quality and a comfortable loudness level. If vocal intensity was outside 70–75 dB, an error signal was displayed after the trial (i.e., “too loud/soft”). Participants were fitted with circum-aural headphones (AKG HSC171) with integrated condenser microphone positioned at 10 cm from the mouth. They were informed that they would hear their voice through the headphones and that sometimes it might sound odd but they were to continue vocalizing regardless. A total of 108 trials were presented under three conditions of equal frequency (i.e., 36 trials each): normal auditory feedback (i.e., no pitch-shift), 100 cent upward pitch-shift of 400 ms duration, and 100 cent downward shift of 400 ms. After the initial five non-perturbed trials, the order of the conditions was randomized. Latency of the perturbation was 12–15 ms and onset of F0 shift randomly varied between 200 and 400 ms, in 50 ms steps, from onset of vocalization.

The apparatus included a Motu Microbook II USB Audio Interface and Behringer Xenyx 502 mixer connected to a Lenovo laptop running PitchPresent software (UTHSCSA Research Imaging Institute, Version Oct 22, 2013) to control timing, direction and magnitude of F0 shifts and recording of vocal responses. Vocalization was recorded at 48 kHz. Auditory feedback was delivered through the headphones at 80–85 dB. The 10 dB gain between voice and feedback channels was used to mask air-born and bone-conducted voice feedback.

#### Data Analysis

**FIGURE 1:**
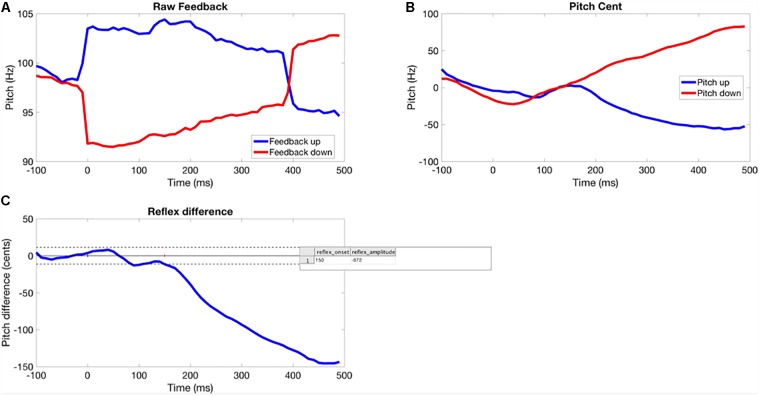
Method for extracting the time course of the perturbation response for each participant. **(A)** Pitch-shifted feedback averaged across perturbation trial. **(B)** Participant’s normalized baseline and opposing responses. **(C)** Averaged difference between response to upward and downward shifts (solid horizontal line showing average baseline response, dotted lines are ±2 SD, response onset is where the signal crosses the 2 SD line for >100 s.

Due to a tendency to falling F0 in the pre-perturbation time window of vocalizations for many participants, a difference method was used to assess F0 trajectory in each trial (see Figure 1). This involved calculating the difference in averaged F0 between up and down perturbation conditions over the time course of each trial for each participant. Differences in pre-perturbation F0 represent noise and the differences between the post-stimulus response to upward and downward shifts represent the pitch-shift response. This will generate response magnitude values roughly twice as large as those reported by Liu et al. (2010). The disadvantage is the inability to determine whether responses to upward and downward shifts differed.

F0 time-series were extracted from each perturbation trial using the interface to PRAAT (Boersma and Weenink, 2010) in the custom software – PitchBrowse (UTHSCSA Research Imaging Institute, Version Oct 22, 2013). This returned an F0 time-series for each perturbation trial with a temporal resolution of 10 ms. Time-series were epoched (-100 to 500 ms) around the time of the onset of the perturbation. Peri-stimulus epochs were then aligned across trials and a sample-wise removal of outliers across trials was performed using the median absolute deviation method (Leys et al., 2013). The time-series were then averaged across trials and the average absolute frequency (Hz) at each sample was changed to cents as a function of pre-perturbation baseline (Kort et al., 2013):

*Cents change* = *100 × [12 × log_2_(pitch response (Hz)/mean pitch frequency of pre-perturbation baseline (Hz))].*

The pitch response to the perturbation was then quantified as the integral of the difference amplitude from the onset of the response until 200 ms after the onset. The onset of the response was determined by a threshold crossing method, whereby variability in pre-perturbation period was used to determine upper and lower bounds for what was regarded as noise. If the response crossed a two-standard deviation bound above or below the pre-perturbation amplitude and remained supra-threshold for more than 100 ms, then this was considered a ‘real’ response. The initial threshold crossing point for this event was taken as the response onset latency.

#### Statistical Analysis

The independent samples *t*-test was used to explore the group effect. For the AOS group, associations between response latency, area under the curve, and AOS and aphasia severity were tested using non-parametric Spearman correlation.

### Results

Two participants were excluded from analysis due to poor quality audio files. There were no missing trials for remaining participants. There was no significant effect of group using independent samples *t*-test [Latency: *t*(1,18) = -0.508, *p* = 0.617, Control Mean = 178.89, SEM = 18.91, AOS Mean = 191.82, SEM = 16.54; Area under the curve: *t*(1,18) = 0.509, *p* = 0.617, Control Mean = -654.11, SEM = 92.58, AOS Mean = -717.09, SEM = 85.50]. For the AOS group, response latency and area under the curve were not significantly correlated with AOS severity (ρ= -0.511, *p* = 0.109 and ρ = 0.284, *p* = 0.398, respectively) or with aphasia severity (*r* = -0.188, *p* = 0.581 and *r* = -0.055, *p* = 0.873, respectively).

### Discussion

Findings for response latency of older healthy adults to the F0 perturbation were similar to those reported by Liu et al. (2010). As predicted, AOS participants demonstrated average latency similar to that of healthy older controls. These results support the hypothesis that feedback mechanisms for this low-level non-segmental auditory parameter, F0 during steady state vowel production, are similar between healthy older adults and adults with AOS. Notably, individuals with AOS typically have a concomitant aphasia. Behroozmand et al. (2018) reported that individuals with aphasia show a reduced response magnitude to F0 perturbation. In that study, response magnitude between 50–150 ms and 250–350 ms post-onset of perturbation was negatively correlated with damage to left posterior language regions (i.e., superior and middle temporal gyri and supramarginal gyrus, respectively) and between 150 and 250 ms with inferior frontal gyrus, centered on *pars orbicularis*. This latter region is more anterior to area 44 (*pars opercularis*) and vPMC traditionally associated with concomitant Broca’s aphasia and AOS.

## Experiment 2

### Methods

#### Participants

Eight of the 12 participants with AOS plus aphasia from Experiment 1 were able to participate in Experiment 2 (five males, three females; *M* = 62.88 years, *SD* = 7.77, range: 54–76; see Table 1). Those excluded were unable to independently produce the stimulus words used in the experiment. A new group of 10 older healthy adults (five males, five females; *M* = 61.5 years, *SD* = 8.5, range: 52–79) were recruited. An additional three older healthy adults (two males, one female) were excluded for failing the audiology screening. Inclusionary criteria are described in Experiment 1. Also, a group of eight adults diagnosed with aphasia only (i.e., no detectable AOS) were recruited as a brain-damaged control (five males, three females; *M* = 59 years, *SD* = 12.7, range: 36–75; see Table 2). AOS and APH groups did not differ on age, years post-stroke, Aphasia Quotient (i.e., aphasia severity), auditory word discrimination using the PALPA, or E_WIL score (Mann–Whitney *U* test, *p*-values 0.161–0.955 and were significantly different for the PVI_WS score, which is an indicator of AOS (*p* = 0.001).

**Table 2:** Demographic and diagnostic testing for participants with aphasia only (APH^∗^) who completed the F1 perturbation study (*N* = 8).

ID^∗^	Sex	Age (years)	Years post-stroke	WAB-AQ (/100)	Aphasia type	PALPA (/72)	E_WIL	PVI_WS
APH32	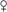	57	5	63	Broca	53	0.52	141.7
APH78	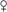	48	3	66	Broca	63	0.05	148.2
APH93	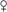	36	2	86.3	Conduction	72	0.10	132.4
APH17	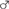	75	4	50	Broca	72	0.05	108.6
APH75	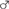	73	4	25.4	Broca	NA	0.45	114.6
APH87	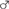	58	18	50.8	Broca	67	0.53	119.0
APH90	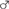	63	1	69.2	Wernicke	66	-0.14	90.7
APH94	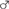	62	4	59.2	Conduction	66	0.44	112.7

	5 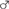	*M* = 59	*M* = 5.1	*M* = 58.7		*M* = 65.6	*M* = 0.25	*M* = 121.0
	3 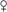	*SD* = 12.7	*SD* = 5.4	*SD* = 17.7		*SD* = 6.5	*SD* = 0.26	*SD* = 18.8


*See notes for Table 1. ^∗^Absence of AOS determined by expert judgment and confirmed by an E_WIL score < 0.17 *and/or* PVI_WS score > 112 (Ballard et al., 2016); Participants. APH17 reported in New et al. (2015) and APH17, 32, and 75 in Ballard et al. (2016).*

#### Apparatus

Audapter, a custom-built MEX-based software (Cai et al., 2008) written in C++ and run within MATLAB (2014b, The Mathworks Inc.), was used to track and shift formant frequencies in real-time. Microphone signal was digitized at a frequency of 48,000 samples/sec and down-sampled to 12,000 samples/sec for real-time processing. Formant frequencies were estimated using an autoregressive linear predictive coding algorithm followed by a dynamic-programming tracking algorithm (Xia and Espy-Wilson, 2000). In this study, the tracked F1 frequencies were mapped to values shifted upward by 30% and then a pole-substituting digital filter converted the formant resonance peaks from their original values to the shifted values. The latency to deliver the perturbed signal was approximately 15 ms, well under the 30 ms threshold for detectable perturbation (Yates, 1963).

Auditory feedback of the participant’s own speech production was delivered through AKG HSC171 circum-aural headphones, with shifted or non-shifted F1 or speech-shaped masking noise depending on the experimental phase. All feedback was delivered at 80 dB SPL to minimize participants’ perception of their own air or bone conducted speech. The fully enclosed design of the headphones provided high ambient noise attenuation and all commented they could not hear their own speech during masking. The condenser microphone integrated with the AKG HSC1716 headphones, 10 cm from the mouth, recorded speech productions.

#### Stimuli

Speech stimuli were five monosyllabic consonant-vowel (CV) words of Australian English: /pε/ (*pear*), /bε/ (*bear*), /kε/ (*care*), /dε/ (*dare*), and /pƆ/ (*paw*). The words *pear, bear*, and *care* were used for training, being presented in baseline and in F1 perturbed conditions. The words *pear, dare*, and *paw* were presented under masking noise to test for adaptation (i.e., *pear*), transfer of the adaptive response to the trained vowel in an untrained phonetic context (*dare*), and for vowel-specificity of any adaptive response (*paw*).

The /ε/ vowel was selected for perturbation because it is a tense mid-vowel covering a large area in F1–F2 space, allowing robust identification of unique spectral peaks of each frequency band and reliable modeling of the target formant with autoregressive analysis. It allows for either opposing or following response to perturbation via raising or lowering of the tongue back (i.e., fall or rise in F1, respectively). An upward perturbation of F1 for /ε/ (i.e., real word *pear*) is associated with lowering of tongue height and shifting of the vowel toward /a/ (i.e., real word *par*; note, the vowel is not rhotacized in Australian English). The vowel /e/ has been used in previous studies, (Ito et al., 2013; Berry et al., 2014; Terband et al., 2014), but in Australian English it is a lax vowel, too brief to allow within-trial tracking of perturbation response.

#### Procedure

The experimental run for each participant consisted of initial instructions on task requirements, then a familiarization phase followed by a four-phase adaptation protocol modeled on the protocol of Villacorta et al. (2007). Initial instructions were to produce a clear vocal quality (i.e., no glottal fry), minimal pitch variation over the vowel, constant speaking volume, and vowel duration of about 500 ms. Participants were given practice trials until they could match the examiner’s model of these response parameters.

The adaptation protocol comprised four phases with a total of 320 trials involving the participant reading aloud each stimulus word as it appeared on a computer screen, at a rate of 1 stimulus per 5 s. The Baseline phase comprised an initial 50 trials, presenting the five stimulus words 10 times each with normal auditory feedback and noise masking randomized, followed by an additional 40 trials with normal auditory feedback. The Baseline established the participant’s habitual F1 in the stimulus words and accounted for any potential variations due to increased vocal intensity with masking (Lombard, 1911). During the subsequent 60-trial Ramp phase, F1 was shifted in an upward direction from 0 to 30% higher than baseline for stimulus words *pear, bear* and *care* only, in increments of 0.5% each trial. This minimized possibility of awareness of the shift. Next, the Hold phase was presented with alternating 15-trial blocks of either 30% F1 perturbed feedback (stimulus words *pear, bear, care*) or noise masking feedback (*pear, dare, paw*). Five blocks of each were provided, for a total of 150 trials. Comparison between the participant’s productions of training words during Baseline and each block of perturbed trials in the Hold phase assessed their sensorimotor compensation, while comparison of the masked productions from Baseline and Hold phases tested for sensorimotor adaptation. Finally, the End phase presented 60 trials under noise masking (stimulus words *pear, dare, paw*) to test for persistence of any adaptation effect. Note that, with a repeated perturbation over consecutive trials, performance in the perturbed trials of the Hold phase actually reflects a combination of compensation to the immediate perturbation and some short-term adaptation to the perturbation from preceding trial blocks. For convenience, we refer to this here as compensation to differentiate it from the longer-term adaptation seen in the absence of perturbation.

Verbal feedback regarding vocal loudness and quality was provided during the four experimental phases only if vocal intensity or quality were notably out of range (e.g., glottal fry). Participant responses were digitally recorded at 48,000 samples/sec for later analysis.

#### Data Analysis

A graphical user interface (GUI) was developed in MATLAB to process each participant’s recorded speech productions. Trials that contained off-target responses (e.g., *pear* substituted for *paw*), large formant-tracking errors (i.e., errors in the timing or accuracy of formant tracking), poor vocal quality or non-speech noises (e.g., cough) were discarded from further analysis. For each recorded stimulus, the most stable artifact-free region of the vowel was manually selected based on visual inspection of the spectrogram. Mean F1 frequency for each extracted vowel segment was then estimated using LPC analysis in MATLAB (Cai et al., 2008). To minimize the occurrence of spurious values, LPC parameters were selected on a per-subject basis. Trials with F1 values below 200 Hz and above 800 Hz were excluded from analysis as they appeared to be outliers. Overall, discarded trials comprised 17/3450 (<1%) for controls, 467/2760 for AOS (16.9%; non-masked trials: 167/1600 or 10.4%, masked trials: 300/1160 or 25.9%), and 642/2760 for APH (23.2%; non-masked trials: 278/1600 or 17.4%, masked trials: 364/1160 or 31.4%). There was no significant difference between the patient groups on number of discarded trials (*p* = 0.159; non-masked trials: *p* = 0.064, masked trials: *p* = 0.556). Data for individual participants are presented in Supplementary Table S1. Reasons for errors included failure to respond within the timeframe, production of the wrong vowel (e.g., producing *pear* for *paw*), or paraphasia (e.g., *peach* for *pear*).

To ensure reliability of the manual vowel selection, 15% of the samples were reanalyzed by a second rater and the original scorer. The intra- and inter-rater reliability and absolute agreement were checked using intraclass correlation coefficient [ICC(3,1)] for three participants each. The intra-rater reliability was ICC(3,1) = 0.83 to 0.97 and the inter-rater reliability was ICC(3,1) = 0.81 to 0.96.

#### Statistical Analysis

The produced trajectories of F1 versus time (trial number) were averaged across the vowel selection. To allow comparisons among participants with differing F1 trajectories, especially in relation to group and sex, each participant’s F1 values were normalized to their mean baseline values as shown in Eq. (1) below, with the dependent variable expressed as a participant’s average response to the perturbation (% F1 change from the Baseline reference of 100%) for each phase or block of trials.

(1) $$Normalized\;F1=\frac{F1}{Mean\;F1\;(Baseline)}\times 100$$

To account for potential vocal intensity differences between masked and unmasked trials, which could influence F1, the Mean F1 obtained from the masked Baseline trials was used to normalize the masked trials in the Hold and End phases while the Mean F1 obtained from the normal feedback Baseline trials was used to normalize the perturbed feedback trials in the Hold phase for each training word. Normality of data was checked and confirmed using probability plots.

To determine the significance of compensatory responses for each group and stimulus type at each block within Hold and End phases, one-sample *t*-tests using the 100% baseline reference value were performed. This analysis established, for each participant group, which blocks within each phase differed significantly from the baseline. To evaluate the differences between groups within each phase, a series of linear mixed model analyses was undertaken as this approach is robust to missing data points in the patient datasets. Factors included Group (three levels), Block (five perturbed and five masked trials blocks for the Hold phase, six masked blocks in the End phase; note that the final block of masking in the Hold phase was continuous with the five blocks in the End phase and so was also included in the End phase analysis). Participant was entered as a repeated factor. LSD *post hoc* testing was undertaken to explore significant effects. Given the exploratory nature of this study, α level was set at 0.05.

### Results

**FIGURE 2:**
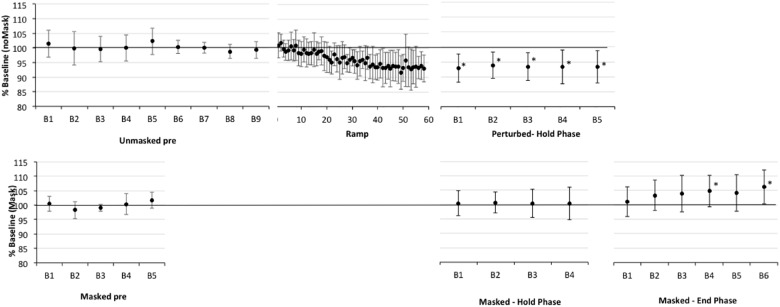
Percent shift in F1, normalized to baseline reference of 100% (solid horizontal line) for older healthy adult participants (CTL). The top panel presents data for production of perturbed words (pear, bear care) in the unperturbed trials in baseline (Pre) and in the F1-shifted trial blocks in Ramp and Hold phases. The bottom panel presents data for production of the word “pear” in masked trials in baseline and in the masked trial blocks of the Hold and End phases. All F1-perturbations were an upward 30% F1 shift. The Ramp phase had no masked trials, the End phase included only masked trials. Error bars show standard deviation. Note that the datapoints in the Ramp are not binned into trial blocks so represent a smaller sample of data than in other phases; also that the first block of masked trials in the End phase can be treated as the final (i.e., fifth) block of masking trials in the Hold phase. Asterisks indicate significant shift in Hold and End phases relative to the baseline reference; also, in Ramp trials 21–60, 37/40 trials are significantly lower than baseline.

**FIGURE 3:**
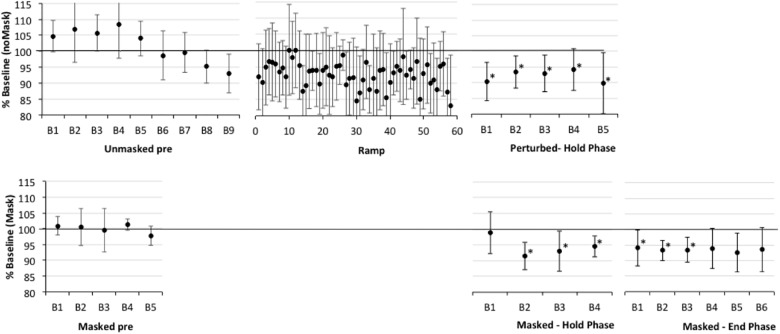
Percent shift in F1, normalized to baseline reference of 100% for older participants with apraxia of speech plus aphasia (AOS). See Figure 2 for details. Asterisks indicate significant shift in Hold and End phases relative to the baseline reference; also, in Ramp trials 21–60, 16/40 trials are significantly lower than baseline.

**FIGURE 4:**
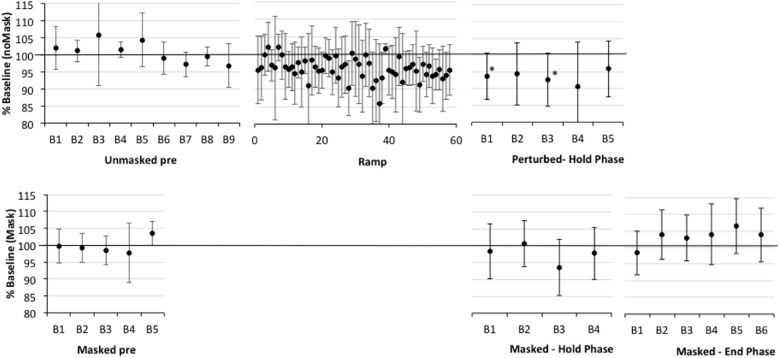
Percent shift in F1, normalized to baseline reference of 100% for older participants with aphasia only (APH). See Figure 2 for details. Asterisks indicate significant shift in Hold and End phases relative to the baseline reference; also, in Ramp trials 21–60, 9/40 trials are significantly lower than baseline.

Performance of the groups is shown in Figures 2–4, Tables 3–5, Supplementary Tables S2–S4, and Supplementary Figure S1 for older controls, AOS and APH groups, respectively.

**Table 3:** Test of fixed effects for compensation in the Hold phase (i.e., perturbed trials) for the dependent variable of percent shift in the first formant of the vowel /ε/, pooled across the words pear /pε/, bear /bε/, and care /kε/.

Source	Numerator df	Denominator df	*F*	*p*
Intercept	1	22.575	254.647	<0.001
Group	2	21.390	0.179	0.837
Block	4	23.000	0.495	0.740
F1 (covariate)	1	22.000	5.107	0.034
Group × Block	8	23.000	0.792	0.615


**Table 4:** Test of fixed effects for adaptation in the Hold phase (i.e., masked trials) for the dependent variable of percent shift in the first formant of the vowel /ε/ in the word pear /pε/.

Source	Numerator df	Denominator df	*F*	*p*
Intercept	1	22.054	12847.379	0.000
Group	2	22.039	4.028	0.032
Block	4	20.488	1.560	0.223
Group × Block	8	20.776	2.948	0.023


**Table 5:** Test of fixed effects for adaptation in the End phase (i.e., masked trials) for the dependent variable of percent shift in the first formant (F1) of the vowel /ε/ in the word pear /pε/.

Source	Numerator df	Denominator df	*F*	*p*
Intercept	1	20.730	133.930	<0.001
Group	2	15.605	6.267	0.010
Block	5	19.280	3.461	0.021
F1 (covariate)	1	20.109	9.130	0.007
Group × Block	10	19.309	2.486	0.041


#### Compensation

First, one-sample *t*-tests considering the perturbed trial blocks of the Hold phase, showed that older controls clearly compensated to the shift in all five blocks relative to baseline (*p*-values of 0.001–0.005), with up to a 7% average drop in F1 (see Figure 2 and Supplementary Table S2). The AOS group also showed significant compensation in all five blocks 1 (*p*-values between 0.003 and 0.047), with up to a 10% average drop in F1 (see Figure 3 and Supplementary Table S3). The APH group showed significant compensation for blocks 1 (*p* = 0.037) and 3 (*p* = 0.034) with up to 9% average drop in F1 (see Figure 4 and Supplementary Table S4).

Comparing across groups, an Unstructured linear mixed model including the fixed effect of Group (three levels), the repeated effect of Block (five levels, perturbed trials only), and the Group by Block interaction, covarying for average F1 on unperturbed trials during the baseline period, was the model with best fit, compared against a first-order regressive covariance structure with or without the covariate (see Table 3). Residuals were normally distributed. However, the main effects of group, block, and the group by block interaction were not significant (also see Figures 2–4 and Supplementary Tables S2–S4).Average vocal intensity (i.e., RMS) in each block was also considered as a covariate and was significant [*F*(1,32.711) = 9.694, *p* = 0.004], but did not alter the outcome of the models.

#### Adaptation in the Hold Phase

One sample *t*-tests considering the perturbed word *pear* in the masked trial blocks of the Hold phase, showed that older controls showed no evidence of adaptation to the shift relative to baseline (*p*-values of 0.591–0.866), with average percent shift ranging between 100.31% (*SD* = 5.63) and 101.06% (*SD* = 5.21)^1^. Consistently, there was no evidence of F1 change for the transfer word (i.e., *dare*) in the first masked trial block (*p* = 0.0804; *M* = 100.37, *SD* = 4.54). The AOS group showed significant adaptation on *pear* for masked trial blocks 2 (*p* = 0.002; *M* = 91.43%, *SD* = 4.40), 3 (*p* = 0.028; *M* = 93.05, *SD* = 6.40), 4 (*p* = 0.022; *M* = 94.05, *SD* = 3.37), and 5 (*p* = 0.032; *M* = 94.01%, *SD* = 5.72) but not for block 1 (*p* = 0.672; *M* = 98.88%, *SD* = 6.63). There was also a significant change in F1 for the transfer word (i.e., dare) in the first masked trial block (*p* = 0.016; *M* = 89.01%, *SD* = 9.88). Similar to controls, the APH group showed no significant adaptation on *pear* (*p*-values of 0.083–0.852), with average percent shift ranging from 93.52 (*SD* = 8.25) to 100.50% (*SD* = 6.83). Consistently, there was no evidence of F1 change for the transfer word (i.e., dare) in the first masked trial block (*p* = 0.282; *M* = 93.62, *SD* = 14.30). No group showed a change in F1 for the control word *paw* in the masked trials of the Hold phase.

Comparing across groups for the perturbed word *pear*, an Unstructured model including the fixed effect of Group (three levels), the repeated effect of Block (five levels), and the Group by Block interaction was the model with best fit, compared against a first-order regressive covariance structure with or without the covariate of average baseline F1 (see Table 4). To ensure that differences in baseline F1 were not driving this result, the average F1 of each participant during the baseline period was considered as a covariate but was not significant [*F*(1,20.731) = 0.000, *p* = 0.983] and inclusion in the Unstructured model did not alter the outcome. Residuals were normally distributed. The main effect of group was significant (*p* = 0.032), as well as the Group by Block interaction (*p* = 0.023), but Block was not significant (*p* = 0.223). LSD pairwise comparisons for group showed that the AOS group differed from controls (*p* = 0.010), with the AOS group tending to have percent shift F1 values below 100% and the controls close to 100%. Compared to the Control group, the AOS group showed significantly lower F1 shift in blocks 2, 3, and 5 (*p* = 0.001, 0.032, 0.022, respectively). Compared to the APH group, the AOS group showed significantly lower F1 shift in block 2 only (*p* = 0.001). No other comparisons were significant. Average vocal intensity (i.e., RMS) in each block was also considered as a covariate but was not significant [*F*(1,32.120) = 1.224, *p* = 0.277] and inclusion in the model did not alter the outcome.

#### Adaptation in the End Phase

One sample *t*-tests considering the perturbed word *pear* in the masked trial blocks of the End phase, showed that older controls showed no evidence of adaptation to the shift relative to baseline with percent shift values at or above 100% (see Supplementary Figure S1 showing individual data for each group). Unexpectedly, two of the final three blocks were significantly above the baseline reference at 104.75 (*SD* = 5.43, *p* = 0.022) and 106.25 (*SD* = 5.93, *p* = 0.009), respectively. For the first masked trial block for transfer word *dare*, there was a similar trend of increasing F1 shift relative to baseline but this was not significant (*p* = 0.086; *M* = 105.42%, *SD* = 8.89). The AOS group showed retention of the Hold phase adaptation effect for *pear* through End blocks 1 (*p* = 0.004; *M* = 93.25%, *SD* = 3.28) and 2 (*p* = 0.011; *M* = 93.46%, *SD* = 4.06). While mean percent shift values did not change across the remaining blocks, ranging from 92.55 to 93.93, some participants did not produce sufficient on-target productions for analysis and there was likely insufficient power to reach significance. For the first block for the transfer word *dare*, the F1 shift approached significance (*p* = 0.054; *M* = 93.32, *SD* = 6.52). Similar to controls, the APH group showed no significant adaptation for *pear* in the End phase (*p*-values of 0.089–0.294), with average percent shift ranging from 102.91 to 106.30. Also there was no change on the transfer word *dare* (*p* = 0.969; *M* = 99.89, *SD* = 6.01). No group showed a change in F1 for the control word *paw* in the masked trials of the End phase.

To explore whether the rising trend in percent change of F1 for older controls may be related to vocal intensity with the extended delivery of masking, we performed Pearson’s correlations. For older controls, vocal intensity (i.e., RMS) was highly correlated with percent shift across blocks (*r* = 0.963, *p* = 0.002). This was not the case for the AOS and APH groups (*r* = -0.017, *p* = 0.974 and *r* = 0.330, *p* = 0.523, respectively). We also explored whether baseline instability (SD of F1) might explain the degree of adaptation (percent change in F1) in the End phase across groups. AOS participants showed significantly more individual variability than controls, with the APH participants being intermediate between these two groups [*F*(2,22) = 5.567, *p* = 0.011; Levene statistic for homogeneity of variance = 5.413, *p* = 0.012 and so the Dunnett T3 *post hoc* test was used: AOS vs. CTL *p* = 0.041, AOS vs. APH *p* = 0.513, APH vs. CTL *p* = 0.239]. Considering the whole participant sample, irrespective of group, there was no significant correlation between individual variability in baseline and degree of adaptation in the End phase (*r* = -0.177, *p* = 0.399); this also was not significant when considering the AOS group alone (*r* = 0.291, *p* = 0.526), though this analysis was under-powered.

Comparing across groups, an Unstructured model including the fixed effect of Group (three levels), the repeated effect of Block (six levels, including the final six consecutive blocks of masking), and the Group by Block interaction, covarying for average F1 during the baseline period, was the model with best fit, compared against a first-order regressive covariance structure with or without the covariate (see Table 5). Residuals were normally distributed. There was a significant effect for group (*p* = 0.010), block (*p* = 0.021), and the group by block interaction (*p* = 0.041).

### Discussion

Auditory perturbation studies were undertaken to determine the integrity of feedback and feedforward speech control processes in healthy older adults and adults with acquired AOS. Given that adults with AOS typically have some degree of co-existing aphasia, we also recruited a group of patients with aphasia only (APH) to test whether any differences in speech motor control might be due to a general effect of neurological damage. It was hypothesized that (a) compensatory feedback responses to unexpected perturbation of a low-level non-segmental auditory parameter, F0 during steady state vowel production, would be similar between healthy older adults and adults with AOS, and (b) with sustained F1 perturbation, older controls and the APH group would demonstrate adaptation that persisted after the perturbation signal was replaced with masking noise, but AOS participants would demonstrate no adaptation. The first hypothesis regarding feedback control was supported. However, the second hypothesis regarding adaptation of feedforward motor programs was not supported: healthy older adults and those with APH showed no clear adaptation within the short time frame tested, while performance of those with AOS suggested adaptation.

#### Compensation

The data from Experiment 1 indicate that individuals with AOS do show immediate compensation to an auditory perturbation of F0, similar to older healthy adults. The magnitude of the F0 compensatory response in both older healthy and AOS participants was similar to that reported by Liu et al. (2010) for healthy older adults (60–73 years). Liu et al. (2010) noted that older adults produce a larger response magnitude, though similar latency, compared with young adults and proposed that this may be due to increasing sensitivity to changes in voice auditory feedback with age or experience. The finding of relatively normal compensation to F0 perturbation in AOS is consistent with work by us and others arguing that the prosodic disturbance in AOS is related to controlling relative durations of speech segments rather than pitch or loudness contrasts. One other adult neurological population, adults with Parkinson’s disease, has been tested with the F0 perturbation task (Liu et al., 2012). In contrast to AOS, Parkinson’s disease is notable for changes in perception and production of the level and variation of their own fundamental frequency and vocal intensity (Duffy, 2013). It is, therefore, not surprising that this group demonstrate abnormal responses to F0 perturbation, with significantly larger response magnitude compared to age-matched older controls.

Recall that performance in the F1 perturbed trials of the Hold phase in Experiment 2 reflects a combination of compensation to the immediate perturbation and some short-term adaptation to the perturbation from preceding trial blocks. We have referred to this as compensation to differentiate it from the longer-term adaptation seen in the absence of perturbation. This experiment suggests that all groups showed some compensation to the F1 perturbation; although, findings for the patient groups should be interpreted with caution due to their baseline variability.

#### Adaptation

Previous work with healthy younger adults (e.g., Villacorta et al., 2007) has shown that repeated exposure to F1 perturbation will result in modification of the feedforward commands (motor program) for the vowel, measured in blocks of masked trials interspersed between perturbed trials, and retention of altered F1 production into subsequent unperturbed trials (i.e., into initial trials of the End phase). We thus expected that older adult controls would adapt to the perturbation. This was not the case. The controls, as well as the older adults with aphasia, but not AOS, showed no difference from baseline F1 in masked trials during each block of the Hold phase and the first block of masked trials in the End phase. Similarly, there was no change in the transfer word *dare* that was only presented under the masking condition. In contrast to healthy older adults and those with APH, the AOS group showed significant adaptation during masked trials in the Hold phase and this persisted into the first blocks of the End phase. The effect was also observed for the transfer word *dare* that was only presented under the masking condition.

The unexpected finding of adaptation to a sustained F1 perturbation in AOS patients but not age-matched healthy controls could be interpreted as follows. In light of this negative finding, it is noteworthy that we found significant adaptation using the exact protocol with healthy young speakers (see Supplementary Figure S2). To account for this difference, we speculate that older controls may possess less “plastic” motor programs that are relatively insensitive, in this brief timeframe, to auditory feedback manipulations compared to younger controls. This has been explored with F1 and F0 perturbations with healthy older adults (Mollaei et al., 2013; Abur et al., 2018, respectively). Abur et al. (2018) ended the perturbation abruptly at the start of the End phase and showed an immediate return to the baseline F0 level at the first data point of the End phase (i.e., an average of the first five trials), which could be interpreted as consistent with our findings of no adaptation under masking. Mollaei et al. (2013) did find adaptation in older healthy adults using an abrupt perturbation onset rather than a ramped onset as used here; one possible explanation for the difference between our finding of no adaptation and the finding of adaptation in their study could be that the abrupt perturbation onset produces a much larger initial auditory error signal that in turn caused greater adaptation than our slowly ramped perturbation onset. Adaptation in older adults has also been studied in the limb system where it appears preserved (Bohm et al., 2015). For example, McCrum et al. (2016) presented a sustained perturbation to young, middle and older healthy females during 18 consecutive right leg swings during walking. While rate of adaptation was slower over the first three steps, the older adults reached the same level of adaptation as younger groups. It is worth noting, however, that with auditory perturbation in the speech system older adults still have access to unperturbed somatosensory feedback, which may attenuate or over-ride their auditory adaptive response. Interestingly, we have shown unilateral deficits in lip and/or tongue somatosensory detection and discrimination in older adults with stroke-related AOS, suggesting diminished feedback through this modality (Etter and Ballard, 2016).

On the surface, the measured adaptation to a sustained F1 perturbation in AOS patients appears at odds with the DIVA model, particularly the model’s prediction that left vPMC damage should impair the readout of auditory targets for ongoing syllables from this region to auditory cortex, which in turn should diminish the ability to detect and correct auditory errors induced by the perturbation. There are several possibilities for reconciling the model with our findings. One possibility is that auditory targets for speech sounds may not emanate from left vPMC as in the DIVA model, instead arising from brain areas not damaged in AOS such as primary motor cortex. A second possibility is that motor programs for speech sounds may not be entirely contained within vPMC, instead being represented elsewhere in the brain such as right hemisphere vPMC or subcortical regions. This would also account for why AOS patients are able to produce speech sequences such as the experimental stimuli used here; if they had completely lost their speech motor programs due to stroke, they should not be able to produce intelligible syllables since feedback control mechanisms are too slow to fluently control speech (Guenther, 2006). Further, left vPMC is typically only partially damaged in AOS. New et al. (2015) reported, on average, damage to only 22% of left vPMC in our cases with AOS who could be scanned, many of whom were included in this study. It should be noted, however, that there was a large amount of inter-subject variability in lesion extent and location for both the AOS and APH groups so these possible neural accounts should be considered speculative.

It is possible that older individuals with AOS have more plastic (malleable) motor programs due to partial damage to left vPMC (New et al., 2015); and that the system in this state is more susceptible to manipulations of auditory feedback. Some ability to adapt speech motor output is in keeping with intervention studies showing that individuals with AOS can modify their speech behaviors within an intervention session and, with intensive practice over days and weeks, can retain these changes after treatment ends (Bislick et al., 2012; Manes and Robin, 2012; Ballard et al., 2015). Here, we tested whether individual variability in baseline, as an indicator of stability, might be associated with degree of adaptation. However, the small sample size precluded a definitive answer. Furthermore, the adaptation paradigm used here differs from typical interventions since AOS treatments typically provide the patient with an external stimulus that indicates the desired production, whereas our adaptation paradigm relies on internal generation of the correct stimulus/target.

We suggest a note of caution in the interpretation of results of Experiment 2, until replication. Visual inspection of the normal-feedback trials in Figure 3 suggests the possibility that AOS participants reduced F1 as the baseline phase progressed (for unknown reasons, not significantly associated with vocal intensity) and simply maintained this lower F1 throughout the remaining perturbed trials of the experiment. Adaptation in the masked trials of the Hold and End phases was measured against baseline masked trials, which did not show the same drop in F1. However, the baseline masked reference was determined from trials presented in the first half of the baseline before the drop in unperturbed F1 was observed. It is possible that F1 would have also dropped in the masking trials, if they had been presented through the second half of this phase. Previous studies have used normal feedback trials in the End phase to show a return to pre-perturbation performance. Repeating this experiment in a new group of patients using a normal feedback End phase would confirm or refute the finding of adaptation in our AOS participants. Also, a larger sample of the performance in the Hold phase would allow statistical comparison of perturbed versus masked trials per group to determine whether the change in F1 is higher for perturbed trials, reflecting a combined compensation and adaptation response (e.g., Villacorta et al., 2007). Again, this could provide confirmatory support for an adaptation response in AOS.

Considering the absence of adaptive response in the APH group, one might have expected abnormal F1 compensation if the sample had been weighted toward patients with posterior lesions and impaired speech perception (e.g., auditory cortex and temporo-parietal junction; Hickok and Poeppel, 2000; Flagmeier et al., 2014). Our mixed sample of posterior and anterior aphasia types, with type relatively balanced across patient groups, would have obscured any specific effects of lesion site. This could be explored with samples specifically selected for lesion location and aphasia type (see Behroozmand et al., 2018 for an investigation of response to F0 perturbation by lesion location in aphasia).

### Limitations and Future Directions

The sample sizes were relatively small, limiting generalizability of the findings. In addition, the number of stimuli and trials presented to participants was reduced, compared with some previous studies (e.g., Villacorta et al., 2007). Terband et al. (2014) also used a shorter protocol in their study with speech-impaired children. This is necessary for impaired populations who find independent word production more challenging than controls and are likely to experience more rapid fatigue. This is one possible reason for the lack of adaptation seen in the older healthy controls and the APH group. Testing compensation and adaptation over multiple sessions may be one way to generate a larger dataset and also explore presence and stability of adaptation over a longer time frame. Further, previous adaptation studies have used non-perturbed trials in the End phase to explore time taken to return to baseline once normal feedback is restored. Here, we chose to mask all trials in the End phase to test how long a potential adaptation effect might persist in the absence of feedback. Performance under a normal-feedback End phase condition would be informative.

It is possible that the AOS and APH groups differed on their ability to perceptually analyze auditory speech input in the F1 perturbation study, as is seen in cases with damage to the temporo-parietal junction. Prior studies have used neural stimulation methods to demonstrate the role of parietal cortex in facilitating or dampening speech adaptation based on somatosensory (Shum et al., 2011) and auditory (Deroche et al., 2017) perturbations. While this may explain the lack of adaptation in the APH group, who may have had parietal damage, the groups did not differ on auditory word discrimination ability completed during diagnostic testing. However, this testing only probed consonant discrimination. A just noticeable difference task for “pear,” manipulating F1 and using each participant’s own voice was attempted here (modeled on that used by Villacorta et al., 2007). However, many stroke participants had difficulty understanding the requirements of the task so that any data were judged invalid. Further, this task measures conscious detection of differences while the perturbation task measures processing that does not require conscious reflection. This limitation should be addressed in future studies.

Finally, the number of discarded trials for the patient groups was undesirable. This is unavoidable in this testing context, where auditory modeling of each target word is not possible. Having only a single target word may reduce the error rates.

## Conclusion

Using auditory perturbation paradigms, we found that AOS participants had normal auditory feedback control for a non-segmental auditory parameter (F0) and displayed motor adaptation to a sustained perturbation of a segmental parameter (F1). The latter finding contrasted with age-matched controls and individuals with aphasia without AOS, who showed no adaptation to the F1 perturbation. These findings suggest that older healthy adults may have less plastic motor programs that resist modification based on sensory feedback, whereas individuals with AOS have less well-developed motor programs due to damage from stroke. Furthermore, they indicate that individuals with AOS can improve their speech motor programs with practice, a capability that is crucial to the success of speech therapies aimed at improving speaking skills in AOS.

The finding of adaptation in the AOS group contrasts with the DIVA model prediction that left vPMC damage resulting in AOS should also impair the readout of auditory targets for ongoing speech sequences, which in turn should impair motor adaptation to a sustained auditory perturbation. Computational modeling, systematically manipulating extent of damage to left hemisphere vPMC/speech sound maps may shed light (e.g., Terband et al., 2009). Methodological differences between this study and those of prior adaptation studies suggest the need for further testing before drawing definitive conclusions.

## Author Contributions

KB, MH, PS, AK, DR, JT, and FG contributed to hypotheses and experimental designs. KB, MH, PS, AK, and AD analyzed the data and setup. KB and AK conducted the experiments and wrote the manuscript. All authors edited the manuscript.

## Conflict of Interest Statement

The authors declare that the research was conducted in the absence of any commercial or financial relationships that could be construed as a potential conflict of interest.

## Footnotes

1 Data for young healthy controls have been collected for a different experiment, using the identical setup, showing replication of the previously reported adaptation effect in the Hold and End phase for this group (see Supplementary Figure S2).
