# Supplementary figures and images for: An Investigation of Compensation and Adaptation to Auditory Perturbations in Individuals With Acquired Apraxia of Speech
Source: Front Hum Neurosci. 2018 Dec 19;12:510. doi: 10.3389/fnhum.2018.00510 (PMC6305734; doi:10.3389/fnhum.2018.00510)

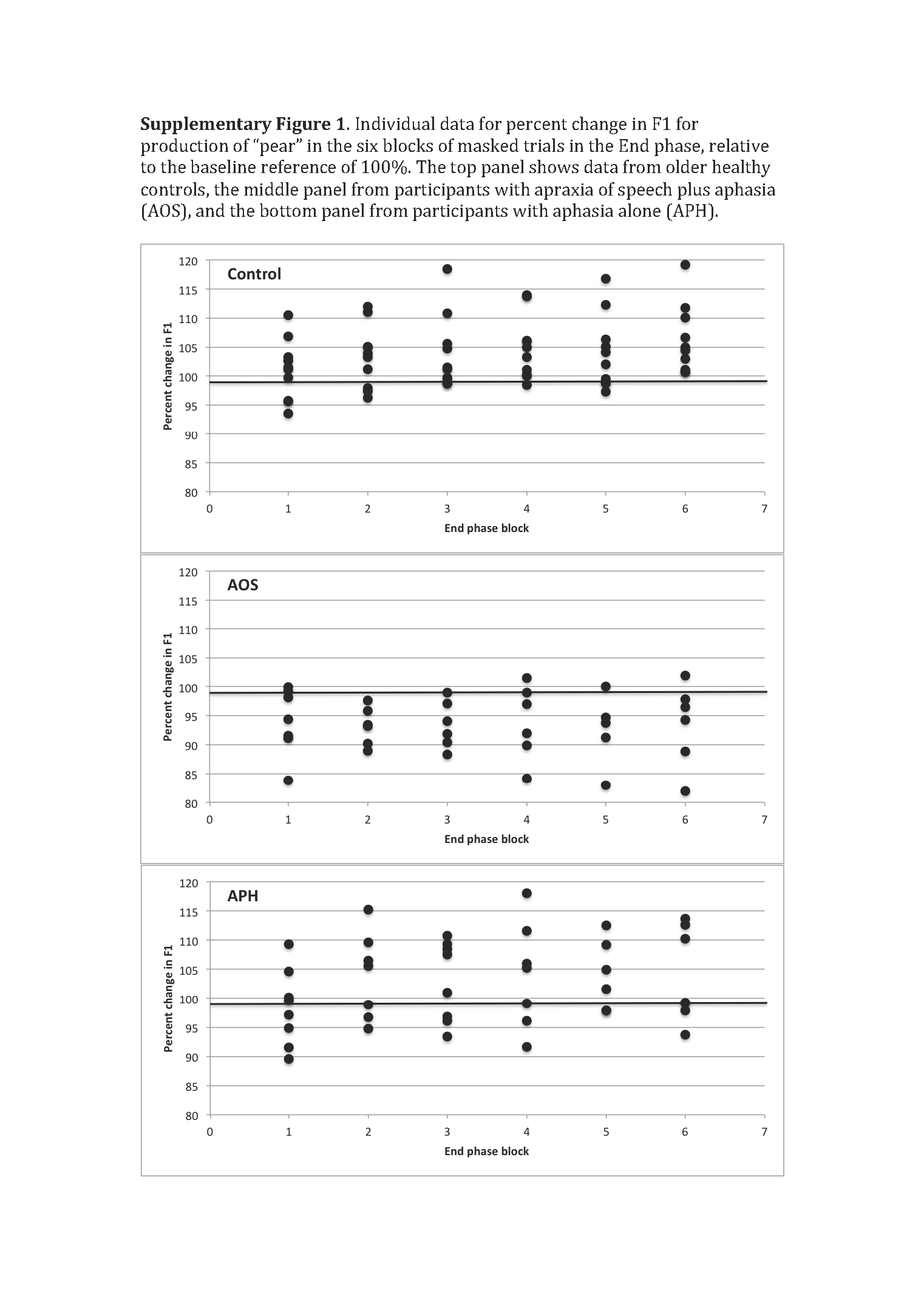

Supplement: Supplementary file 1 [file Image_1.tiff]

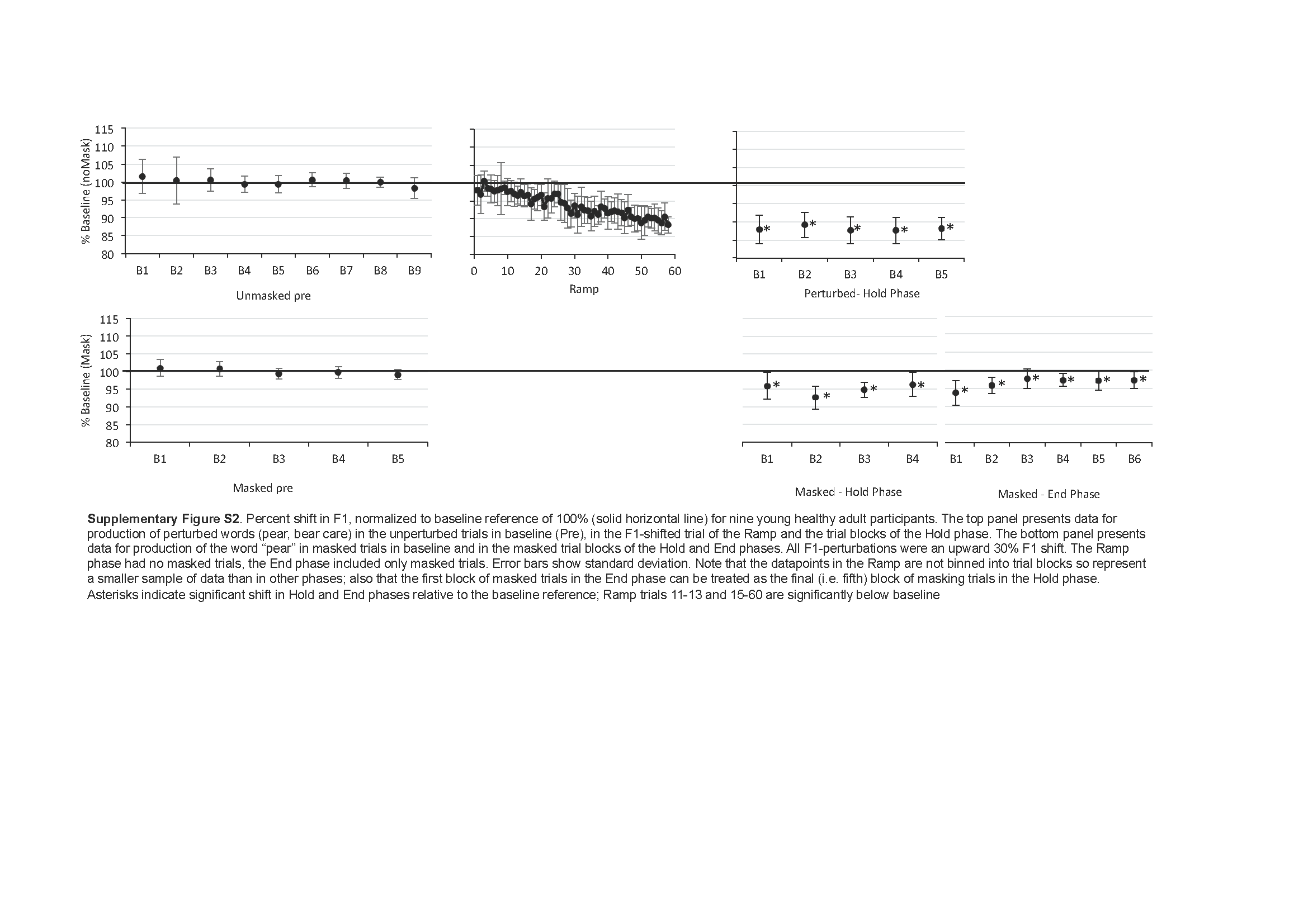

Supplement: Supplementary file 2 [file Image_2.tiff]
