# Supplementary material for: An Investigation of Compensation and Adaptation to Auditory Perturbations in Individuals With Acquired Apraxia of Speech
Source: Front Hum Neurosci. 2018 Dec 19;12:510. doi: 10.3389/fnhum.2018.00510 (PMC6305734; doi:10.3389/fnhum.2018.00510)
Supplement: Supplementary file 3 [file Table_1.docx]

**Table S1.** Number of discarded trials in the F1 perturbation study for the older control (CTL), apraxia of speech (AOS), and aphasia (APH) groups by experimental phase.

| **Participant** | **Group** | **Baseline** | | **Ramp** | **Hold** | | **End** | **Total** |
| --- | --- | --- | --- | --- | --- | --- | --- | --- |
|  |  | Not masked (n = 65) | Masked  (n = 25) | Not masked  (n = 60) | Not masked  (n = 75) | Masked^a^  (n = 60) | Masked  (n = 60) | (n = 345) |
| 1 | CTL | 0 | 0 | 0 | 13 | 0 | 0 | 13 |
| 2 | CTL | 0 | 0 | 0 | 0 | 0 | 0 | 0 |
| 3 | CTL | 1 | 0 | 0 | 0 | 0 | 0 | 1 |
| 4 | CTL | 0 | 0 | 0 | 0 | 0 | 0 | 0 |
| 5 | CTL | 0 | 0 | 0 | 0 | 0 | 0 | 0 |
| 6 | CTL | 0 | 0 | 1 | 0 | 2 | 0 | 3 |
| 7 | CTL | 0 | 0 | 0 | 0 | 0 | 0 | 0 |
| 8 | CTL | 0 | 0 | 0 | 0 | 0 | 0 | 0 |
| 9 | CTL | 0 | 0 | 1 | 0 | 0 | 0 | 0 |
| 10 | CTL | 0 | 0 | 0 | 0 | 0 | 0 | 0 |
| AOS22 | AOS | 16 | 19 ^b^ | 13 | 13 | 37 | 47 ^b^ | 145 |
| AOS24 | AOS | 0 | 0 | 1 | 0 | 2 | 3 | 6 |
| AOS49 | AOS | 0 | 0 | 0 | 3 | 4 | 0 | 7 |
| AOS60 | AOS | 16 | 6 | 23 | 12 | 17 | 38 | 112 |
| AOS77 | AOS | 1 | 0 | 0 | 7 | 12 | 1 | 21 |
| AOS79 | AOS | 8 | 12 | 2 | 13 | 27 | 40 | 102 |
| AOS88 | AOS | 7 | 2 | 7 | 20 | 16 | 13 | 65 |
| AOS92 | AOS | 1 | 0 | 2 | 2 | 4 | 0 | 9 |
| APH17 | APH | 14 | 13 | 16 | 19 | 18 | 18 | 98 |
| APH32 | APH | 8 | 7 | 13 | 10 | 16 | 53 ^c^ | 107 |
| APH75 | APH | 25 | 16 | 12 | 29 | 45 | 39 ^d^ | 166 |
| APH78 | APH | 4 | 8 | 0 | 0 | 0 | 0 | 12 |
| APH87 | APH | 28 | 11 | 27 | 12 | 41 ^d^ | 41 ^d^ | 160 |
| APH90 | APH | 0 | 0 | 3 | 11 | 3 | 5 | 22 |
| APH93 | APH | 0 | 0 | 0 | 0 | 3 | 0 | 3 |
| APH94 | APH | 16 | 6 | 13 | 18 | 14 | 7 | 74 |
| ^a^ The final 15-trial masked block of the Hold phase is consecutive with the masking trials of the End phase and is counted in the End phase here.  ^b^ Baseline: 6 productions were available for calculating the baseline reference for words with the perturbed vowel /ε/; no productions were available for the control vowel /Ͻ/; End: 25 errors were non-responses and 12 were paraphasic substitution of the word “drum”.  ^c^ abandoned the task part way through the End phase trials.  ^d^ Produced the word “pear” for most trials. | | | | | | | | |
|  |  |  |  |  |  |  |  |  |
|  |  |  |  |  |  |  |  |  |
|  |  |  |  |  |  |  |  |  |
|  |  |  |  |  |  |  |  |  |
|  |  |  |  |  |  |  |  |  |
|  |  |  |  |  |  |  |  |  |
|  |  |  |  |  |  |  |  |  |
|  |  |  |  |  |  |  |  |  |
