# Supplementary material for: An Investigation of Compensation and Adaptation to Auditory Perturbations in Individuals With Acquired Apraxia of Speech
Source: Front Hum Neurosci. 2018 Dec 19;12:510. doi: 10.3389/fnhum.2018.00510 (PMC6305734; doi:10.3389/fnhum.2018.00510)
Supplement: Supplementary file 4 [file Table_2.docx]

**Table S2.** One sample t-tests (two tailed) for the older control (CTL) group comparing F1 percent shift relative to the 100% baseline reference across all phases of the experiment (* p < .05).

|  | Mean | SD | t | df | p | Mean Difference | 5% CI of the Difference | |  |  |
| --- | --- | --- | --- | --- | --- | --- | --- | --- | --- | --- |
|  |  |  |  |  |  |  | Lower | Upper |  |  |
| **Compensation: HOLD phase (pear, bear, care; F1 perturbed)** | | | | | | | | |  |  |
| Block 1 | 92.95 | 4.76 | -4.68 | 9 | **.001*** | -7.05 | -10.45 | -3.64 |  |  |
| Block 2 | 93.82 | 4.42 | -4.41 | 9 | **.002*** | -6.18 | -9.35 | -3.01 |  |  |
| Block 3 | 93.39 | 4.62 | -4.52 | 9 | **.001*** | -6.61 | -9.92 | -3.30 |  |  |
| Block 4 | 93.32 | 5.70 | -3.70 | 9 | **.005*** | -6.68 | -10.76 | -2.60 |  |  |
| Block 5 | 93.34 | 5.40 | -3.90 | 9 | **.004*** | -6.66 | -10.52 | -2.80 |  |  |
| **Adaptation: HOLD phase (pear only, masked)** | | | | | | | | |  |  |
| Block 1 | 100.48 | 4.33 | .35 | 9 | .734 | .48 | -2.62 | 3.58 |  |  |
| Block 2 | 100.64 | 3.61 | .56 | 9 | .591 | .64 | -1.94 | 3.21 |  |  |
| Block 3 | 100.48 | 4.91 | .31 | 9 | .766 | .48 | -3.04 | 3.99 |  |  |
| Block 4 | 100.31 | 5.63 | .17 | 9 | .866 | .31 | -3.72 | 4.34 |  |  |
| Block 5 | 101.06 | 5.21 | .65 | 9 | .534 | 1.06 | -2.66 | 4.79 |  |  |
| **Adaptation: END phase (pear only; masked)** | | | | | | | | |  |  |
| Block 1 | 103.29 | 5.35 | 1.94 | 9 | .084 | 3.29 | -.54 | 7.11 |  |  |
| Block 2 | 103.90 | 6.39 | 1.93 | 9 | .086 | 3.90 | -.67 | 8.47 |  |  |
| Block 3 | 104.75 | 5.43 | 2.77 | 9 | **.022*** | 4.75 | .87 | 8.64 |  |  |
| Block 4 | 104.10 | 6.33 | 2.05 | 9 | .071 | 4.10 | -.43 | 8.63 |  |  |
| Block 5 | 106.25 | 5.93 | 3.33 | 9 | **.009*** | 6.25 | 2.01 | 10.49 |  |  |
| **Transfer: HOLD phase (dare only; masked)** | | | | | | | | |  |  |
| Block 1 | 100.37 | 4.54 | .26 | 9 | .804 | .37 | -2.88 | 3.61 |  |  |
| Block 2 | 101.01 | 5.84 | .54 | 9 | .600 | 1.01 | -3.17 | 5.18 |  |  |
| Block 3 | 100.55 | 5.17 | .34 | 9 | .743 | .55 | -3.14 | 4.25 |  |  |
| Block 4 | 100.82 | 5.51 | .47 | 9 | .649 | .82 | -3.12 | 4.76 |  |  |
| Block 5 | 100.92 | 6.29 | .44 | 8 | .672 | .92 | -3.91 | 5.76 |  |  |
| **Transfer: END phase (dare only; masked)** | | | | | | | | |  |  |
| Block 1 | 105.42 | 8.89 | 1.93 | 9 | .086 | 5.42 | -.94 | 11.78 |  |  |
| Block 2 | 103.58 | 5.58 | 2.03 | 9 | .073 | 3.58 | -.41 | 7.58 |  |  |
| Block 3 | 104.94 | 8.65 | 1.81 | 9 | .104 | 4.94 | -1.25 | 11.13 |  |  |
| Block 4 | 104.34 | 6.43 | 2.14 | 9 | .061 | 4.34 | -.25 | 8.94 |  |  |
| Block 5 | 104.75 | 7.21 | 2.08 | 9 | .067 | 4.75 | -.41 | 9.91 |  |  |
| **Control: HOLD phase (paw only, masked)** | | | | | | | | |  |  |
| Block 1 | 99.32 | 9.01 | -.24 | 9 | .817 | -.68 | -7.13 | 5.77 |  |  |
| Block 2 | 96.70 | 9.94 | -1.05 | 9 | .321 | -3.30 | -10.42 | 3.81 |  |  |
| Block 3 | 97.10 | 9.68 | -.95 | 9 | .368 | -2.90 | -9.83 | 4.02 |  |  |
| Block 4 | 97.88 | 9.62 | -.70 | 9 | .503 | -2.12 | -9.01 | 4.76 |  |  |
| Block 5 | 97.11 | 9.07 | -1.01 | 9 | .340 | -2.89 | -9.38 | 3.60 |  |  |
| **Control: END phase (paw only, masked)** | | | | | | | | |  | **13.4007** |
| Block 1 | 102.60 | 15.10 | .54 | 9 | .600 | 2.60 | -8.21 | 13.40 |  |  |
| Block 2 | 98.88 | 11.86 | -.30 | 9 | .772 | -1.12 | -9.60 | 7.36 |  |  |
| Block 3 | 98.70 | 13.95 | -.30 | 9 | .775 | -1.30 | -11.28 | 8.68 |  |  |
| Block 4 | 100.91 | 14.02 | .20 | 9 | .843 | .91 | -9.12 | 10.94 |  |  |
| Block 5 | 101.32 | 12.49 | .33 | 9 | .746 | 1.32 | -7.62 | 10.25 |  |  |
