# Supplementary material for: An Investigation of Compensation and Adaptation to Auditory Perturbations in Individuals With Acquired Apraxia of Speech
Source: Front Hum Neurosci. 2018 Dec 19;12:510. doi: 10.3389/fnhum.2018.00510 (PMC6305734; doi:10.3389/fnhum.2018.00510)
Supplement: Supplementary file 5 [file Table_3.docx]

**Table S3.** One sample t-tests (two tailed) for the apraxia of speech (AOS) group comparing F1 percent shift relative to the 100% baseline reference across all phases of the experiment (* p < .05).

|  | Mean | | | SD | | t | df | p | Mean Difference | | 5% CI of the Difference | | | | |  |  |
| --- | --- | --- | --- | --- | --- | --- | --- | --- | --- | --- | --- | --- | --- | --- | --- | --- | --- |
|  |  | | |  | |  |  |  |  | | Lower | | Upper | | |  |  |
| **Compensation: HOLD phase (pear, bear, care; F1 perturbed)** | | | | | | | | | | | | | | | |  |  |
| Block 1 | | 90.42 | 6.13 | | -4.42 | | 7 | **.003*** | -9.58 | -14.70 | | -4.45 | | |  |  |  |
| Block 2 | | 93.49 | 5.19 | | -3.55 | | 7 | **.009*** | -6.51 | -10.85 | | -2.17 | | |  |  |  |
| Block 3 | | 93.01 | 5.86 | | -3.38 | | 7 | **.012*** | -6.99 | -11.90 | | -2.09 | | |  |  |  |
| Block 4 | | 94.28 | 6.74 | | -2.40 | | 7 | **.047*** | -5.72 | -11.35 | | -0.09 | | |  |  |  |
| Block 5 | | 89.83 | 9.83 | | -2.93 | | 7 | **.022*** | -10.17 | -18.38 | | -1.95 | | |  |  |  |
| **Adaptation: HOLD phase (pear only, masked)** | | | | | | | | | | | | | |  |  |  |  |
| Block 1 | | 98.88 | 6.63 | | -.446 | | 6 | .672 | -1.12 | -7.25 | | 5.02 | | |  |  |  |
| Block 2 | | 91.43 | 4.40 | | -5.16 | | 6 | **.002*** | -8.57 | -12.64 | | -4.50 | | |  |  |  |
| Block 3 | | 93.05 | 6.40 | | -2.87 | | 6 | **.028*** | -6.95 | -12.86 | | -1.03 | | |  |  |  |
| Block 4 | | 94.50 | 3.37 | | -3.65 | | 4 | **.022*** | -5.50 | -9.69 | | -1.32 | | |  |  |  |
| Block 5 | | 94.01 | 5.72 | | -2.77 | | 6 | **.032*** | -5.99 | -11.28 | | -0.70 | | |  |  |  |
| **Adaptation: END phase (pear only; masked)** | | | | | | | | | | | | | |  |  |  |  |
| Block 1 | | 93.25 | 3.28 | | -5.04 | | 5 | **.004*** | -6.75 | -10.19 | | -3.31 | | |  |  |  |
| Block 2 | | 93.46 | 4.06 | | -3.94 | | 5 | **.011*** | -6.54 | -10.80 | | -2.28 | | |  |  |  |
| Block 3 | | 93.93 | 6.44 | | -2.31 | | 5 | .069 | -6.07 | -12.83 | | 0.69 | | |  |  |  |
| Block 4 | | 92.55 | 6.24 | | -2.67 | | 4 | .056 | -7.45 | -15.19 | | 0.30 | | |  |  |  |
| Block 5 | | 93.57 | 7.12 | | -2.21 | | 5 | .078 | -6.43 | -13.91 | | 1.04 | | |  |  |  |
| **Transfer: HOLD phase (dare only; masked)** | | | | | | | | | | | | | |  |  |  |  |
| Block 1 | | 89.01 | 9.88 | | -3.15 | | 7 | **.016*** | -10.99 | -19.25 | | -2.74 | | |  |  |  |
| Block 2 | | 91.19 | 7.96 | | -2.93 | | 6 | **.026*** | -8.81 | -16.16 | | -1.45 | | |  |  |  |
| Block 3 | | 90.55 | 9.39 | | -2.47 | | 5 | .057 | -9.45 | -19.30 | | 0.41 | | |  |  |  |
| Block 4 | | 90.18 | 8.91 | | -2.20 | | 3 | .115 | -9.82 | -24.00 | | 4.37 | | |  |  |  |
| Block 5 | | 89.98 | 11.72 | | -2.09 | | 5 | .090 | -10.02 | -22.32 | | 2.28 | | |  |  |  |
| **Transfer: END phase (dare only; masked)** | | | | | | | | | | | | | |  |  |  |  |
| Block 1 | | 93.32 | 6.52 | | -2.51 | | 5 | .054 | -6.68 | -13.52 | | 0.17 | | |  |  |  |
| Block 2 | | 93.79 | 7.05 | | -2.16 | | 5 | .083 | -6.21 | -13.60 | | 1.19 | | |  |  |  |
| Block 3 | | 98.02 | 8.87 | | -0.55 | | 5 | .609 | -1.98 | -11.29 | | 7.33 | | |  |  |  |
| Block 4 | | 95.01 | 8.18 | | -1.36 | | 4 | .244 | -4.99 | -15.15 | | 5.17 | | |  |  |  |
| Block 5 | | 92.90 | 10.31 | | -1.69 | | 5 | .152 | -7.10 | -17.92 | | 3.71 | | |  |  |  |
| **Control: HOLD phase (paw only, masked)** | | | | | | | | | | | | | |  |  |  |  |
| Block 1 | | 90.94 | 16.34 | | -1.36 | | 5 | .233 | -9.06 | -26.21 | | 8.09 | | |  |  |  |
| Block 2 | | 93.60 | 11.36 | | -1.38 | | 5 | .226 | -6.40 | -18.33 | | 5.52 | | |  |  |  |
| Block 3 | | 97.26 | 12.76 | | -0.53 | | 5 | .621 | -2.74 | -16.13 | | 10.65 | | |  |  |  |
| Block 4 | | 92.64 | 10.93 | | -1.51 | | 4 | .207 | -7.36 | -20.93 | | 6.21 | | |  |  |  |
| Block 5 | | 98.42 | 10.50 | | -0.37 | | 5 | .727 | -1.58 | -12.61 | | 9.44 | | |  |  |  |
| **Control: END phase (paw only, masked)** | | | | | | | | | | | | | |  | | | **13.4007** |
| Block 1 | | 98.48 | 15.28 | | -0.24 | | 5 | .817 | -1.52 | -17.56 | | 14.52 | | |  |  |  |
| Block 2 | | 99.91 | 11.24 | | -0.02 | | 5 | .985 | -0.09 | -11.89 | | 11.71 | | |  |  |  |
| Block 3 | | 101.02 | 17.73 | | 0.14 | | 5 | .893 | 1.02 | -17.59 | | 19.63 | | |  |  |  |
| Block 4 | | 95.66 | 15.83 | | -0.67 | | 5 | .531 | -4.34 | -20.95 | | 12.26 | | |  |  |  |
| Block 5 | | 96.08 | 14.56 | | -0.66 | | 5 | .539 | -3.92 | -19.20 | | 11.37 | | |  |  |  |
