# Supplementary material for: An Investigation of Compensation and Adaptation to Auditory Perturbations in Individuals With Acquired Apraxia of Speech
Source: Front Hum Neurosci. 2018 Dec 19;12:510. doi: 10.3389/fnhum.2018.00510 (PMC6305734; doi:10.3389/fnhum.2018.00510)
Supplement: Supplementary file 6 [file Table_4.docx]

**Table S4.** One sample t-tests (two tailed) for the aphasia (APH) group comparing F1 percent shift relative to the 100% baseline reference across all phases of the experiment (* p < .05).

|  | Mean | | | SD | | | t | | | df | | | p | | | Mean Difference | | | 5% CI of the Difference | | | |  |  |  |
| --- | --- | --- | --- | --- | --- | --- | --- | --- | --- | --- | --- | --- | --- | --- | --- | --- | --- | --- | --- | --- | --- | --- | --- | --- | --- |
|  |  | | |  | | |  | | |  | | |  | | |  | | | Lower | Upper | | |  |  |  |
| **Compensation: HOLD phase (pear, bear, care; F1 perturbed)** | | | | | | | | | | | | | | | | | | | | | | |  |  |  |
| Block 1 | 93.68 | | 6.97 | | | -2.57 | | 7 | | | **.037*** | | | -6.32 | | | -12.15 | | | | | -.050 | |  |  |
| Block 2 | 94.34 | | 9.21 | | | -1.74 | | 7 | | | .126 | | | -5.66 | | | -13.37 | | | | | 2.04 | |  |  |
| Block 3 | 92.65 | | 7.94 | | | -2.62 | | 7 | | | **.034*** | | | -7.35 | | | -13.99 | | | | | -0.72 | |  |  |
| Block 4 | 90.71 | | 13.26 | | | -1.98 | | 7 | | | .088 | | | -9.29 | | | -20.38 | | | | | 1.79 | |  |  |
| Block 5 | 95.86 | | 8.37 | | | -1.40 | | 7 | | | .204 | | | -4.14 | | | -11.14 | | | | | 2.86 | |  |  |
| **Adaptation: HOLD phase (pear only, masked)** | | | | | | | | | | | | | | | | | | | | | | |  |  |  |
| Block 1 | 98.22 | | 8.06 | | | -0.62 | | 7 | | | .552 | | | -1.78 | | | -8.52 | | | | | 4.96 | |  |  |
| Block 2 | 100.50 | | 6.83 | | | 0.20 | | 6 | | | .852 | | | 0.50 | | | -5.82 | | | | | 6.82 | |  |  |
| Block 3 | 93.52 | | 8.25 | | | -2.08 | | 6 | | | .083 | | | -6.48 | | | -14.11 | | | | | 1.15 | |  |  |
| Block 4 | 97.71 | | 7.71 | | | -0.84 | | 7 | | | .428 | | | -2.29 | | | -8.74 | | | | | 4.15 | |  |  |
| Block 5 | 98.35 | | 6.56 | | | -0.71 | | 7 | | | .499 | | | -1.65 | | | -7.14 | | | | | 3.84 | |  |  |
| **Adaptation: END phase (pear only; masked)** | | | | | | | | | | | | | | | | | | | | | | |  |  |  |
| Block 1 | 103.89 | | 7.45 | | | 1.38 | | 6 | | | .217 | | | 3.89 | | | -3.00 | | | | | 10.78 | |  |  |
| Block 2 | 102.91 | | 6.89 | | | 1.20 | | 7 | | | .271 | | | 2.91 | | | -2.85 | | | | | 8.68 | |  |  |
| Block 3 | 103.96 | | 9.11 | | | 1.15 | | 6 | | | .294 | | | 3.96 | | | -4.47 | | | | | 12.39 | |  |  |
| Block 4 | 106.30 | | 8.21 | | | 2.03 | | 6 | | | .089 | | | 6.30 | | | -1.30 | | | | | 13.89 | |  |  |
| Block 5 | 103.79 | | 8.12 | | | 1.24 | | 6 | | | .263 | | | 3.79 | | | -3.71 | | | | | 11.30 | |  |  |
| **Transfer: HOLD phase (dare only; masked)** | | | | | | | | | | | | | | | | | | | | | | |  |  |  |
| Block 1 | 93.62 | 14.30 | | | -1.18 | | | | 6 | | | .282 | | | -6.38 | | | -19.61 | | | 6.84 | | |  |  |
| Block 2 | 99.83 | 3.53 | | | -0.12 | | | | 5 | | | .910 | | | -0.17 | | | -3.87 | | | 3.53 | | |  |  |
| Block 3 | 97.06 | 6.62 | | | -0.99 | | | | 4 | | | .377 | | | -2.94 | | | -11.16 | | | 5.28 | | |  |  |
| Block 4 | 95.69 | 12.49 | | | -0.77 | | | | 4 | | | .484 | | | -4.31 | | | -19.82 | | | 11.20 | | |  |  |
| Block 5 | 94.63 | 7.51 | | | -1.60 | | | | 4 | | | .185 | | | -5.37 | | | -14.70 | | | 3.95 | | |  |  |
| **Transfer: END phase (dare only; masked)** | | | | | | | | | | | | | | | | | | | | | | |  |  |  |
| Block 1 | 99.89 | 6.01 | | | -0.04 | | | | 4 | | | .969 | | | -0.11 | | | -7.57 | | | 7.35 | | |  |  |
| Block 2 | 102.66 | 6.22 | | | 0.96 | | | | 4 | | | .393 | | | 2.66 | | | -5.06 | | | 10.39 | | |  |  |
| Block 3 | 106.89 | 10.49 | | | 1.47 | | | | 4 | | | .216 | | | 6.89 | | | -6.14 | | | 19.92 | | |  |  |
| Block 4 | 102.44 | 3.96 | | | 1.38 | | | | 4 | | | .240 | | | 2.44 | | | -2.47 | | | 7.36 | | |  |  |
| Block 5 | 96.05 | 13.55 | | | -0.72 | | | | 5 | | | .507 | | | -3.95 | | | -18.17 | | | 10.26 | | |  |  |
| **Control: HOLD phase (paw only, masked)** | | | | | | | | | | | | | | | | | | | | | | |  |  |  |
| Block 1 | 97.97 | 11.15 | | | -0.48 | | | | 6 | | | .647 | | | -2.03 | | | -12.34 | | | 8.28 | | |  |  |
| Block 2 | 92.99 | 6.78 | | | -2.53 | | | | 5 | | | .052 | | | -7.01 | | | -14.13 | | | 0.11 | | |  |  |
| Block 3 | 93.57 | 11.26 | | | -1.40 | | | | 5 | | | .221 | | | -6.43 | | | -18.24 | | | 5.39 | | |  |  |
| Block 4 | 93.95 | 8.37 | | | -1.62 | | | | 4 | | | .182 | | | -6.05 | | | -16.45 | | | 4.35 | | |  |  |
| Block 5 | 94.12 | 13.85 | | | -1.04 | | | | 5 | | | .346 | | | -5.88 | | | -20.41 | | | 8.66 | | |  |  |
| **Control: END phase (paw only, masked)** | | | | | | | | | | | | | | | | | | | | | | |  | | **13.4007** |
| Block 1 | 101.75 | 8.59 | | | 0.46 | | | | 4 | | | .672 | | | 1.75 | | | -8.92 | | | 12.41 | | |  |  |
| Block 2 | 103.43 | 10.16 | | | 0.76 | | | | 4 | | | .492 | | | 3.43 | | | -9.19 | | | 16.06 | | |  |  |
| Block 3 | 104.81 | 10.73 | | | 1.10 | | | | 5 | | | .322 | | | 4.81 | | | -6.45 | | | 16.07 | | |  |  |
| Block 4 | 105.26 | 9.56 | | | 1.23 | | | | 4 | | | .286 | | | 5.26 | | | -6.61 | | | 17.12 | | |  |  |
| Block 5 | 102.62 | 6.43 | | | 1.00 | | | | 5 | | | .364 | | | 2.62 | | | -4.13 | | | 9.37 | | |  |  |
